# Understanding and Defining Young People's Involvement and Under‐Representation in Mental Health Research: A Delphi Study

**DOI:** 10.1111/hex.14102

**Published:** 2024-06-14

**Authors:** Rachel Perowne, Sarah Rowe, Leslie Morrison Gutman

**Affiliations:** ^1^ UCL Centre for Behaviour Change, Division of Psychology and Language Sciences University College London London UK; ^2^ Division of Psychiatry University College London London UK

**Keywords:** coproduction, diversity, involvement, mental health research, patient and public involvement, representation, young people

## Abstract

**Introduction:**

The mental health of young people (aged 16–25 years) is a growing public health concern in the United Kingdom due to the increasing numbers of young people experiencing mental health difficulties, with many not in contact with mental health services. To design services that meet the needs of all young people, a diversity of young people must be involved in mental health research, beyond being participants. This Delphi study aimed to identify different types of ‘involvement’ and to define and describe ‘under‐representation’ in young people's involvement in mental health research.

**Methods:**

Twenty‐seven experts in young people's mental health research completed a series of online questionnaires. The experts were academic researchers, patient and public involvement (PPI) professionals and young ‘experts by experience’. Round 1 generated panellists' views on ‘involvement’ and ‘under‐representation’. Round 2 summarised panellists' responses from Round 1 and sought consensus (minimum 70% agreement) in nine question areas. Round 3 validated the findings of the previous rounds.

**Results:**

Consensus was achieved in eight out of nine areas, resulting in a matrix (with definitions) of the different types of young people's involvement in mental health research, from being advisors to involvement ambassadors. The findings generated an agreed‐upon definition of under‐representation, an identification of when in the research process there is under‐representation and the characteristics of the young people who are under‐represented. Experts further agreed on demographic data that should be collected to improve reporting on involvement.

**Conclusions:**

This study adds to our understanding of involvement and under‐representation in the context of young people's mental health research through expert consensus. It provides a practical resource for researchers considering involving young people in the research process and suggests the data that should be collected to improve reporting on the diversity of the young people involved.

**Patient and Public Contribution:**

A research oversight group of five young people advised on this study. They contributed throughout the project—from endorsing the research question to commenting on the findings and dissemination. Two of the group reviewed all participant materials and piloted the initial questionnaire.

## 1 Introduction

Recent increases in mental health problems among young people are a major public health concern in the United Kingdom [1]. In 2023, 23.3.% of 17‐ to 19‐year olds in England, and 21.7% of 20‐ to 25‐year olds, had a ‘probable mental health disorder’ [2], compared to 16.9% of 16‐ to 23‐year olds in 2021 [3]. However, it is estimated that three in four young people with clinically significant mental ill‐health symptoms are not in contact with mental health services [4], for reasons including service inaccessibility and cultural stigma around mental health, especially among certain groups [5, 6, 7]. Young people are not a homogeneous group [8] and experience mental health issues differently, with important implications for diagnosis and treatment [9]. There are sociodemographic driven differentials in the prevalence of different mental health disorders, their presentation, and in young people's use of mental health services [1]. For example, rural young people can have difficulty accessing services [10], ethnic minority young people can be put off engaging with services due to low trust in health professionals [9] and male role expectations can prevent young men from seeking help [11].

There is growing recognition that, to be effective and inclusive, mental health systems need to be ‘culturally responsive’ and reflect all voices and perspectives [12]. Mental health research is critical in providing the evidence to achieve this [4], but needs to include diverse young people in the research that informs the knowledge base—both as participants, and through active involvement activities [13, 14, 15]. Meaningfully involving young people provides integrity to research, enhancing relevance and responsiveness through young people's voices [15, 16], especially ‘disadvantaged’ or ‘marginalised’ groups [16, 17]. It can aid recruitment, improve study design, and increase the impact of research [18, 19, 20]. Young people's insights in data analysis can result in more robust studies and enhance the translation of findings into practice [15, 21], leading to improved services [22]. It also benefits the young people involved, empowering them by giving them a voice, teaching them new skills, and providing a supportive community [20, 23].

While youth involvement is becoming more mainstream, the extent of involvement varies widely [8, 24]. Many models of involvement exist in the literature. For example, the ‘Ladder of Coproduction’ [25] in health and social care, ‘Hart's Ladder of Participation’ [26], Lundy's ‘Children's Participation Model’ [27] and Shier's ‘Pathways to Participation’ Model [28], all reflecting young people's involvement across different contexts (see Wilson et al. [14] for further examples) and the ‘McCain model of Youth Engagement’ which describes Youth‐Adult Partnership in young people's mental health [29]. These models differ in their terminology, definitions and scope of involvement. In a complex and evolving landscape, and given the plethora of terminology describing involvement, a shared understanding of young people's involvement in mental health research is required to optimise involvement, and reporting, through identifying both good practice and areas for improvement [21, 23].

Having diversity in the youth involved in research leads to more valid, reliable and representative results [30], and is important to young people themselves [31], but it does not always happen in practice [24, 32]. The limited evidence that exists suggests that groups such as ethnic and sexual minority young people, and those living in rural areas, are under‐represented in involvement in mental health research [7, 14, 33, 34, 35, 36]. However, young people's involvement is often not reported on in detail [37]. Many studies either do not report on the sociodemographics of the young people involved, representing a gap in knowledge and evidence in this area, or the sample involved is not diverse [14, 23, 24].

This study aims to elucidate conceptions of young people's ‘involvement’ and ‘under‐representation’ in mental health research to fill these gaps. It uses a Delphi design, with a diverse group of experts in young people's mental health, to address these questions and generate new insights [38, 39]. The Delphi approach has been applied elsewhere in health research to establish research priorities and frameworks [39, 40, 41], and within mental health research specifically, to define key concepts [42].

The study aims to address the research questions:

RQ1. What are the different types of involvement of young people in mental health research and how can these be described?

RQ2. How is under‐representation in the involvement of young people in mental health research defined, when in the process does it occur and which groups are under‐represented?

## 2 Materials and Methods

### 2.1 Design

**Figure 1:**
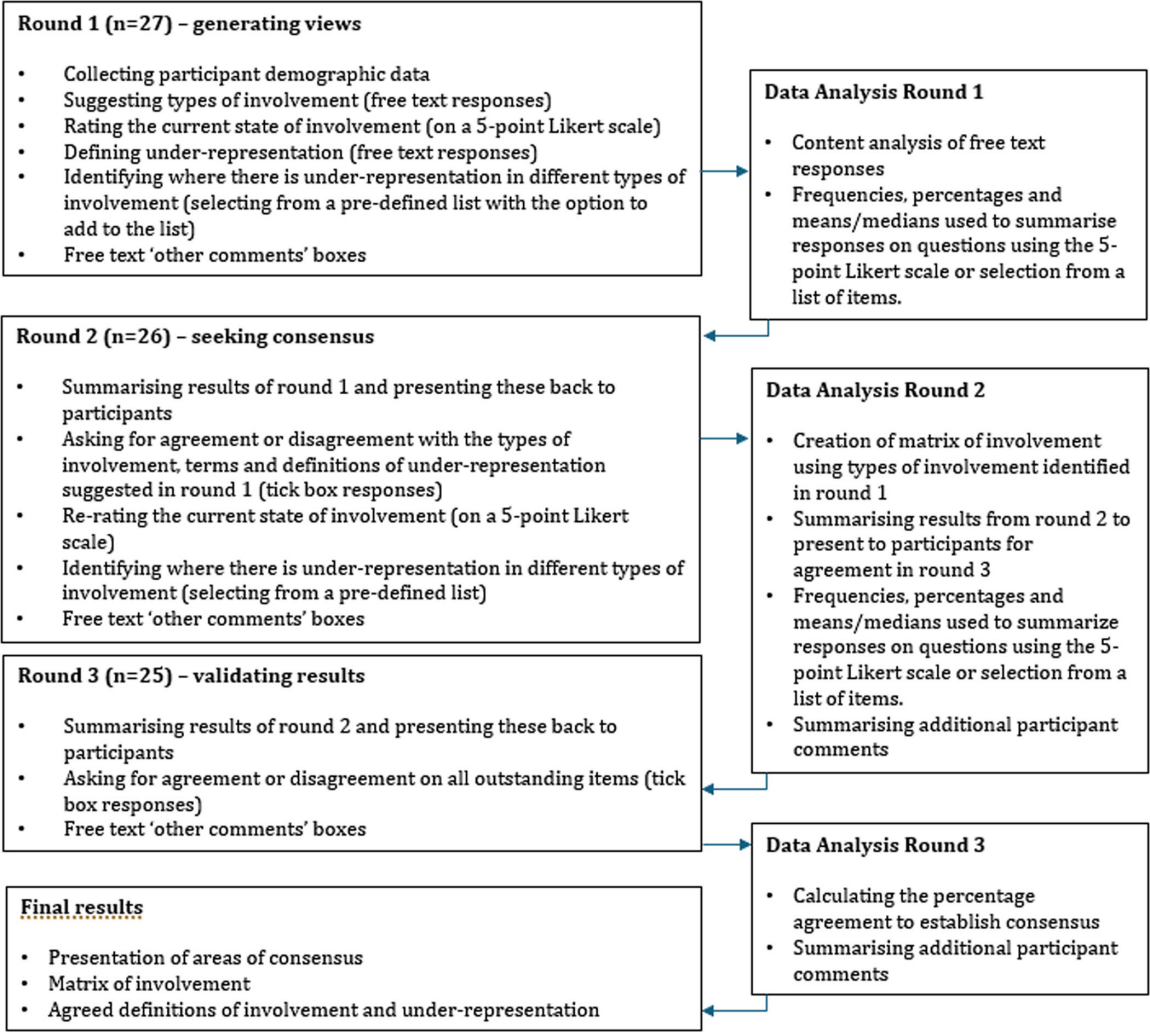
Delphi process flow chart.

A Delphi design based on McPherson et al. [38] and Brady [43] was used. Online questionnaires allowed multiple experts, across different regions of the United Kingdom and contexts, to take part, anonymously and without the power dynamics that can affect study designs, such as focus groups [38, 39, 41]. This is particularly relevant when young people are involved [15, 16, 44]. Three categories of experts were included to ensure a diversity of knowledge and experience [36, 41, 42]; academic researchers and patient and public involvement (PPI) professionals, with experience of involving young people in mental health research and young people (aged between 16 and 25 years, in line with the UK Government's recent Green Paper, ‘Transforming Children and Young People's Mental Health Provision’) [45] with expertise through previous involvement in mental health research. Experts completed three iterative rounds of questionnaires, with responses to each round informing the next [37, 43] (Figure 1), a process which provides rigour, control and validity to the findings [43].

### 2.2 Sample and Recruitment

A purposive, snowballing sampling strategy was used, with adverts circulated through relevant academic, public and third‐sector networks [36]. Direct email approaches were also made to experts in the field, through hand‐searching academic papers, online directories and referrals. Participants were offered a £25 Amazon voucher for taking part. At least six experts from each group were sought to achieve the minimum recommended panel size [38, 46] while remaining within resource constraints [47]. Interested parties were emailed participant information and completed an online consent form, confirming their eligibility. They were also asked to outline their experience, in at least one of the three expert groupings, in terms of organisational affiliations, description of relevant projects, authored papers and length of experience in the field (see Appendix S1) [48].

Of 27 participants, five were PPI professionals, eight young people with experience of being involved in mental health research, 10 academic researchers, one PPI professional who had also been involved in research as a young person and three had experience in all three categories. Job roles included PPI facilitator, research fellow, coproduction lead, research assistant, peer researcher and Young People's Advisory Group (YPAG) member. Participants provided their ethnicity and sex/gender identity through an open‐text box. Twelve participants identified as White British/English. Twenty participants were female, six male/cis male and one nonbinary. As the aim of the study was to understand ‘under‐representation’, demographic categories were not defined a priori and participants were simply asked whether they considered themselves to be from an under‐represented group (see Table 1) and which aspects of their identity this related to. Fifty‐nine percent (*n* = 16) responded yes to this question with reported characteristics including ethnicity (*n* = 9), sexual orientation (*n* = 5), neurodiversity (*n* = 5), religion/belief (*n* = 3), socioeconomic status (*n* = 3), disability (*n* = 2), mental health condition (*n* = 2), sex/gender (*n* = 1) and immigration status (*n* = 1).

**Table 1:** Participant demographics.

Participant ID	Ethnicity	Sex or gender identity	Type of expert	Self‐identified as from an under‐represented group?
1	White British	Female	PPI professional	No
2	Asian British	Female	PPI professional	Yes
3	White British	Female	Academic researcher	No
4	White British	Female	Young person	Yes
5	White British	Male	Academic researcher	No
6	White British	Cis male	PPI professional	Yes
7	White British	Female	Academic researcher PPI Professional Young person	No response provided
8	White British	Female	Academic researcher	Yes
9	Mixed–Black African and White	Female	Young person PPI professional	Yes
10	White British	Male	Young person	Yes
11^a^	British African‐Asian	Female	Young person	Yes
12	Asian	Male	Academic researcher	Yes
13	Indigenous native American	Female	Academic researcher	Yes
14	Indian	Female	Young person	Yes
15	White British	Female	Academic researcher	No
16	British	Female	Academic researcher PPI Professional Young person	Yes
17	White Asian	Female	Young person	Yes
18	White	Nonbinary	Young person	Yes
19^b^	White British	Female	PPI professional	Yes
20	White English	Man	Academic researcher	No
21	Asian	Female	Academic researcher PPI Professional Young person	Yes
22	White non‐British	Female	Young person	Possibly
23	Arab	Female	Young person	Yes
24	Asian Indian	Female	Academic researcher	Possibly
25	White British	Female	Academic researcher	No
26	White other	Male	PPI professional	No
27	White European	Female	Academic researcher	No

*Note:* All responses provided were free text rather than selection from a list.

a Participated in Rounds 1 and 2 but not Round 3.

b Participated in Round 1 only.

### 2.3 Procedures

Data collection took place over 11 weeks with online questionnaires issued to participants every 3–4 weeks. Round 1 generated experts' views, using mainly open‐ended questions. Round 2 sought consensus, by providing experts with summaries of Round 1 responses and asking them to rerate items which had not achieved consensus, and in Round 3 experts validated the results of the previous rounds, leading to the final results.

#### 2.3.1 Round 1

The Round 1 questionnaire comprised demographic information and questions in nine areas: types of young people's involvement in mental health research, effectiveness of involvement, definitions of under‐representation, terms for under‐representation, diversity within involvement, under‐represented groups, under‐representation in different stages and roles within research and finally categories for socio‐demographic data collection. Questions were mainly open‐ended to obtain experts' free‐flowing ideas [41], with a small number of questions using a 5‐point Likert rating scale or selection of items from an a priori list, with the option to add items or comments [42, 48, 49]. Questions were devised through reviewing recent relevant literature, including a number of significant systematic reviews [13, 14, 15, 16, 22, 24] as well as grey literature (such as NIHR and Department of Health reports) [18, 45, 50, 51] and identifying gaps in knowledge in the field [42, 43]. Questions included ‘Please list the different types of involvement you are aware of that young people could have in mental health research’ (free text response), ‘How well do you think young people from diverse backgrounds and identities are currently involved in mental health research?’ (rating on a 5‐point Likert scale, plus free text comments) and ‘What socio‐demographic information should we collect from young people involved in mental health research?’ (selection of items from an a priori list plus the option to add items) (see Appendix S1 for the full questionnaire). The questionnaire was piloted by at least one expert from each of the three categories and reviewed by the second and third authors before being finalised.

#### 2.3.2 Round 2

The second round questionnaire aimed to achieve consensus in each question area by providing a summary of first‐round responses, for panellists to either agree or disagree with, or to revise their previous ratings in view of these summaries.

#### 2.3.3 Round 3

The final questionnaire validated results from the previous rounds. Participants were asked to agree a matrix, generated through data from previous rounds, depicting a summary of the levels, activities and roles within young people's involvement. Additional summary statements were provided for agreement, with space for additional comments.

### 2.4 Analyses

Data were collected and aggregated using Microsoft Forms and exported to Microsoft Excel for analysis. Content analysis was used to analyse responses to open‐ended questions [46]. Participant comments were broken down into items of data, preserving participants' wording wherever possible [37, 43]. Similar items were grouped together, analysed and a universal label and description created to form themes. The number of participants mentioning a particular item of data or theme and the emphasis placed on it by participants was recorded. For questions requiring a rating or item selection, the means and medians or frequency and percentages of each option selected were calculated. This information was reported back to participants in subsequent rounds, along with a summary of themes identified in, and quotes from, participant comments [46]. This allowed participants to see how their responses compared to the group. Consensus was defined a priori as achieving a minimum of 70% agreement [40, 46, 52, 53]. Where consensus was achieved on an item, participants were not asked to rerate this item in subsequent rounds but were provided feedback on the result and asked for further comments. Where consensus was not achieved, the question was carried over to the subsequent round with a summary of participant ratings and comments to inform reratings [54].

### 2.5 Young People's Involvement

Young people participated as both experts and, additionally, a separate group of young people was consulted through a Young Researchers' Oversight Group (YROG). This was a diverse group of five young people, from within an existing YPAG, who responded to an expression of interest. YROG members were between the ages of 16 and 18 years. Two identified as male and three female. White, Asian British, Arab and ‘multiple ethnic groups’ were represented. World views included Christian, Muslim and Atheist or Agnostic. Sexual orientations included heterosexual, gay and questioning. Levels of parental education ranged from GCSE to Doctoral degree (or equivalents) and the young people were all in full‐time education. All five were born in the United Kingdom, but four had a parent born outside the United Kingdom and for one, English was not their first language. The group included young people who had experienced mental health problems, disability or long‐term health conditions and who were neurodiverse. One had caring responsibilities. The young people lived in a variety of locations—urban, suburban, and rural.

The YROG met roughly every 2 months to advise on the project. They discussed and endorsed the research questions and study plans. Consultation was via Zoom meetings and electronically through the young people commenting on documents. Meetings were held in the evenings to suit young people's schedules and included the young people's YPAG co‐ordinator for safeguarding reasons. Two group members piloted the first round questionnaire and reviewed all participant information. The YROG reviewed and commented on the study findings and contributed to dissemination plans. Reimbursement was in line with recommended NIHR rates [55] and cost approximately £500 including attendance at meetings and work outside these. The young people's involvement is reported in this paper using the GRIPP2 checklist for the transparent and consistent reporting of patient and public involvement activities in research [35] (see Appendix S2).

### 2.6 Ethics

The study was registered with the University's Data Protection Officer and received approval from UCL Ethics Committee (20293/002).

## 3 Results

### 3.1 Response Rates

Twenty‐seven participants took part in Round 1, with 26 of those continuing to Round 2 and 25 in Round 3, representing an overall drop‐out rate of 7.4%.

### 3.2 Consensus

Consensus was reached on at least one item in six of the nine question areas in Round 2. A further two areas reached consensus in Round 3, leading to consensus being achieved overall for items in eight of nine question areas (shown in Table 2).

Detailed results following the final round are reported below. Relevant participant quotes from all rounds are provided, labelled using the participant's identification number followed by the round from which the quote comes (e.g., P1R2 is participant 1, round 2).

**Table 2:** Summary of results and consensus by round.

Topic	Results of Round 1	Results of Round 2	Results of Round 3
Expert participants	27	26 (see Table 1 for details)	25 (see Table 1 for details)
Response rate	100%	96.3%	92.6%
Types of young people's involvement	12 types of involvement suggested	Consensus (between 73.1% and 100%) was achieved that 10 of the 12 types suggested were types of involvement. 2 types of involvement (‘participants’ and ‘dissemination’) did not reach consensus.	Consensus achieved on matrix of involvement (80%) and between 80% and 100% on the definitions of different involvement types
Effectiveness of involvement of young people in mental health research (ratings out of 5)	Mean rating 2.4 Median rating 2	No consensus on a single rating Mean rating 2.5 Median rating 2.5	Consensus achieved (92%) that young people's involvement in mental health research can be described as ‘somewhat poor’
Definitions of under‐representation	27 suggested definitions. These were analysed and summarised into 15 different definitions for Round 2	2 of the 15 definitions achieved consensus (76.9%). These definitions were combined to produce a single definition.	Consensus (100%) achieved on definition of under‐representation
Preferred terms for under‐representation	14 alternative terms suggested	Consensus was not achieved on any term. The most selected term for under‐representation received 54% agreement	Consensus not achieved. Experts provided final comments on the summary provided
Diversity within involvement of young people in mental health research (ratings out of 5)	Mean rating 2 Median rating 2	No consensus on a single rating Mean rating 1.9 Median rating 2	Consensus achieved (at 92%) that diversity in young people's involvement in mental health research can be described as ‘poor’
Characteristics of young people under‐represented in mental health research	17 characteristics were identified	5 characteristics of young people achieved consensus (between 73.1% and 96.2%) 12 characteristics did not achieve consensus	Experts provided final comments on the summary provided
Stages of the research process in which there is under‐representation	Experts selected from 8 stages of the research process with no additional stages suggested	6 (of 8) stages of the research process achieved consensus (between 80.1% and 96.2%) as having under‐representation of young people 2 stages of the research process did not achieve consensus	Experts provided final comments on the summary provided
Types of roles where there is under‐representation	Experts suggested roles (including coresearchers, coproduction and young advisors)	Consensus achieved (84.6%) for all roles	Experts provided final comments on the summary provided
Categories of sociodemographic data that should be collected from young people involved in mental health research	Experts suggested a further 10 categories in addition to 9 included in the questionnaire	Consensus (between 70.1% and 96.2%) was reached on 17 (out of the 19) categories 2 categories of data did not achieve consensus	Experts provided final comments on the summary provided

### 3.3 Involvement

**Figure 2:**
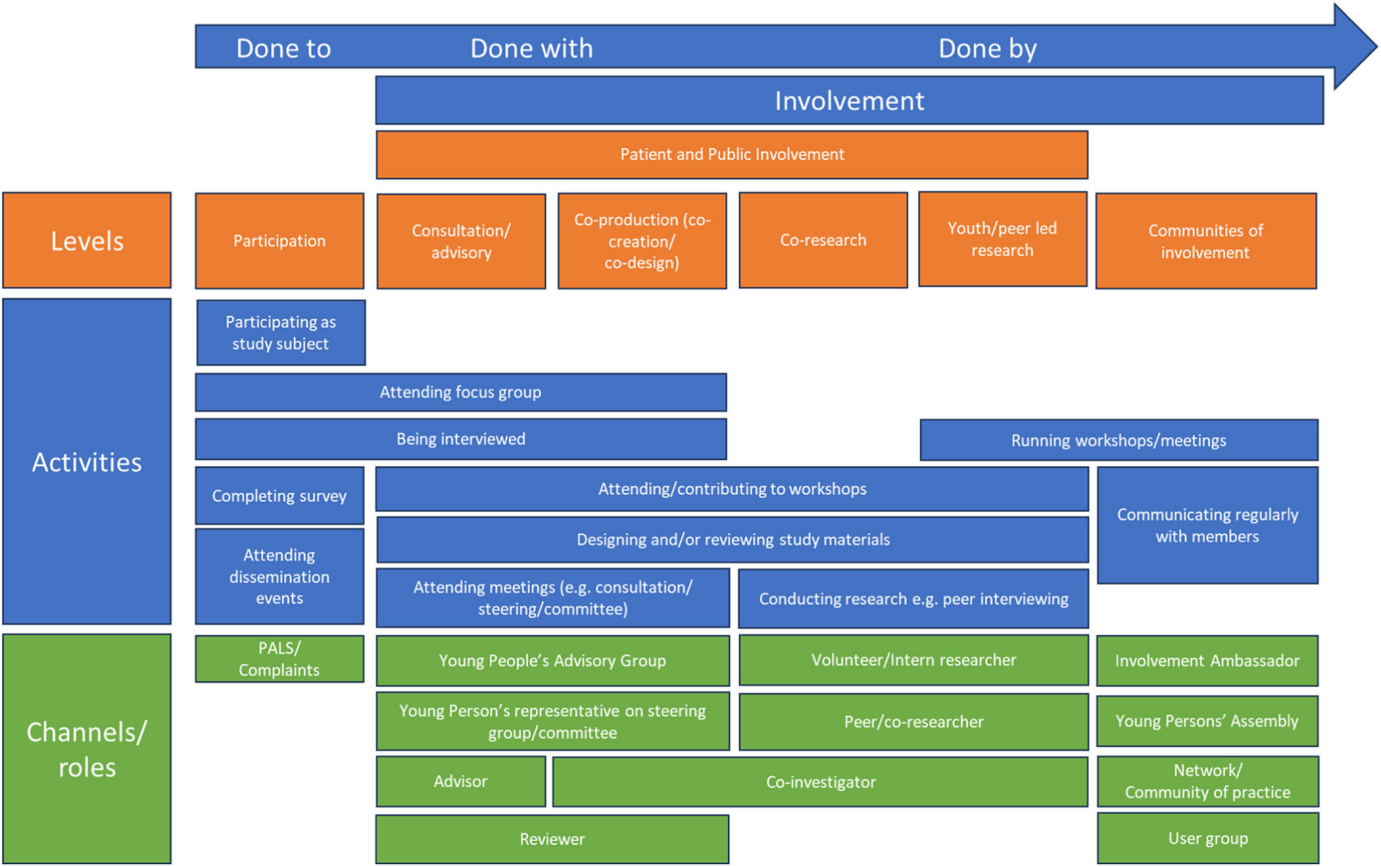
Matrix of Young People's Involvement in mental health research. PALS, patient advice and liaison service.

Participants reached consensus on different types of involvement, resulting in a Matrix of Young People's Involvement (MYPI) in mental health research (Figure 2) with accompanying definitions of types of involvement (Table 3).

**Table 3:** Types of involvement, definitions identified by expert panellists.

Type of involvement	Consensus that this is involvement?	Agreed definition
Type 1: Young People's Advisory Group	Yes	A group or panel for consulting young people, sometimes with lived experience of mental health issues, convened to inform research activity. Young people share their views and experiences as well as providing advice and feedback to researchers to make meaningful contributions to improve research and inform decision making, either on a project specific, or regular and ongoing, basis.
Type 2: Coproduction (including codesign and cocreation)	Yes	A process of active involvement and collaboration with young people, either as a collective or individually, throughout the research process (from inception and creation to implementation and dissemination)—starting from scratch/a blank page. Following the 5 coproduction principles, young people are equal and reciprocal partners, gaining benefit themselves and working alongside researchers, having a say about how the research is done and being part of decision‐making.
Type 3: Consultation or advisory role	Yes	Young people's views and expertise are sought in relation to a project either on a one‐off or ongoing basis. Input is often limited in scope and the young people's advice may or may not be acted upon. In this sense, the young people are not equal and reciprocal partners in the research in the same way as in coproduction.
Type 4: Coresearch/peer‐research	Yes	Young people are very actively involved in key stages of the research process, bringing lived experience to the research and conducting research themselves, with the supervision of a senior researcher. A slightly more involved role than coproduction. This could be done through an internship, bursary or a voluntary or paid opportunity.
Type 5: Patient and Public Involvement	Yes	An umbrella term for coproduction and codesign which involves activities that add value or knowledge to the project through seeking the advice and opinion of young people, who would be affected by the research, on a piece of mental health research.
Type 6: Young person/peer‐led involvement and research	Yes	Young people, with lived experience, take charge of all aspects of the research process: leading, having ownership, meaningful involvement and making decisions across all stages of the research process. Researchers provide supervision and support but there is reciprocity and power is equally shared with the young people.
Type 7: Reviewer	Yes	Young people review or evaluate research documents, potentially as part of a panel of reviewers, and provide feedback and recommendations.
Type 8: Young people's involvement network/community of practice	Yes	A youth‐focused approach to involvement which can make different types of involvement more meaningful. Networks or communities of young people who have an interest in being involved in research are developed. Some networks act as a vehicle for young people to find out about opportunities or they can be groups that meet regularly for young people to direct the involvement process and decide which research questions should be addressed, and types of involvement to engage in. They are a resource for young people to learn about new opportunities to get involved.
Type 9: Young people as participants	No	Young people participate in research, as a study subject or service‐user.
Type 10: Young persons' representative on a scientific committee	Yes	For example, young people being representatives on a thesis committee or steering committee.
Type 11: Involvement ambassadors	Yes	Young people (from communities who are all too often easily ignored) are set up as involvement champions in health care research.
Type 12: Dissemination	No	Feeding back results of research to participants and the broader community.

*Note:* A more detailed table with example involvement activities and illustrative participant quotes is provided in Appendix S3.

#### 3.3.1 MYPI in Mental Health Research

Participants agreed (by consensus of 80%) that the MYPI was an accurate representation of young people's involvement in mental health research. The MYPI is organised as a continuum of involvement from left to right, with ‘participation’, which panellists did not agree was a type of involvement, on the left, representing young people being ‘done to’ as passive subjects of a research process. Levels, such as ‘consultation’ and ‘coproduction’, represent research ‘done with’ young people as partners. ‘Young people/peer‐led research’, which is research ‘done by’ young people, appears on the right of the continuum, representing greater levels of involvement. Each type of involvement is accompanied by a definition agreed through participant consensus (Table 3). The activities and channels/roles sit underneath the relevant level of involvement. For example, within ‘communities of involvement’, ‘running workshops/meetings’ might be an activity within a ‘young persons’ assembly’. Other activities, such as ‘attending focus groups’, straddle more than one level of involvement.

#### 3.3.2 Effectiveness of Young People's Involvement in Mental Health Research

Participants rated the effectiveness of young people's involvement in mental health research in the United Kingdom. The median, and mean, response was 2.5 (out of five) which panellists agreed by consensus (at 92%) could be summarised as ‘somewhat poor’. One participant who disagreed commented that ‘services like MQ research and McPin foundation… are now being so much more involving [of] young people—but more awareness of this is needed!’ (P4R3).

Participants were also asked ‘How well do you think young people from diverse backgrounds and identities are currently involved in mental health research?’ The mean rating was 1.9 (out of 5) and the median was 2 which experts agreed by consensus (at 92%) was ‘poor.

### 3.4 ‘Under‐Representation’

#### 3.4.1 Defining Under‐Representation

Of the 16 terms suggested for ‘under‐representation’ in involvement, and voted on by panellists, no single term reached consensus, but ‘under‐representation’, remained most selected (by 54% of panellists (14 out of 26). One panellist commented ‘I like that the term doesn't put the onus on those who have been unable to get their voices heard at the “table”’. (P1R1). However, limitations of this term included ‘it could be vague as a “group of under‐represented people” like a minority group, is not necessarily the same as someone being “under‐represented”.’ (P17R1).

A definition for under‐representation was arrived at through panellists each creating a definition which was then voted on. The final (100%) consensus definition was:

Low involvement and insufficient diversity and representation of young people with lived experience from different backgrounds and demographics, such as marginalised populations, different socioeconomic backgrounds, sexuality, ethnicity or those with intersectional identities.

Under‐represented characteristics were agreed through consensus, as; minority ethnic, lower socioeconomic status, disability (including learning disability), asylum seekers/immigrants/refugees and young people whose first language is not English. Twelve characteristics did not achieve consensus: LGBTQIA+, lower academic/educational level, gender (young men), neurodiversity, care experience, carers, religion, serious mental illness, rural location, gypsy/Roma/travellers, international students and inpatients). However, panellists acknowledged that ‘More work needs to be done to include young people from all backgrounds’ (P2R3).

#### 3.4.2 Where Does Under‐Representation Happen?

Panellists agreed (85%) that there was under‐representation in all types of involvement; from advisory to co‐research roles. One panellist observed, ‘YPAGs have the best representation, but are still unrepresentative of most young people. Youth‐led communities of practice and champions can support changing this’ (P7R2). There was consensus that under‐representation occurred at the beginning (setting research priorities, commissioning and securing funding and designing and planning studies) and the end (dissemination, knowledge translation, and evaluation) of the research process but not in the data collection and analysis stages. One panellist, P17R3 (a young person) felt that ‘it is important to have young people's input on HOW the data may be analysed and disseminated … but I don't think they would need to actually do the analysis themselves’.

#### 3.4.3 What Data Should be Collected From Young People to Monitor Under‐Representation?

Panellists agreed that data on age, sex, gender, ethnicity, religion and belief, disability, neurodiversity, socioeconomic status, carer status, location (urban/rural), education (attainment and setting), migration status, language, care experience, sexuality, mental health diagnosis and medical conditions, should be collected when reporting the diversity of young people involved in mental health research. Family mental health history and relationship status did not reach consensus. One participant noted that ‘Several of these categories should only be collected if relevant to the research project’ adding that ‘collecting more thorough data … will help us to better understand which groups are not being heard’ (P5R2).

#### 3.4.4 Outcome and Impacts of PPI

The involvement of the YROG resulted in changes to the study design and participant materials including: improving the recruitment advert for a younger audience, defining certain terms within the participant information, improving accessibility through a mobile‐enabled questionnaire and making the language across all participant documentation ‘Plain English’. The YROG commented on the findings and results of the study. They suggested improving the description of the MYPI to aid reader understanding, provided their perspectives on the effectiveness of young people's involvement in mental health research and identified their preferred terms and definition for under‐representation. Their views are reflected in the results and discussion.

## 4 Discussion

Through an iterative process, this Delphi study explored ‘involvement’ and ‘under‐representation’ of young people in mental health research.

### 4.1 Involvement

The MYPI produced adds to the knowledge base by representing and providing clarity on the wide range of involvement of young people taking place in mental health research. It provides additional levels and extra detail to other models and is underpinned by expert‐developed definitions. The types of involvement included are consistent with the NIHR definition of involvement in research as ‘carried out “with” or “by” members of the public rather than “to”, “about” or “for” them’ and ‘an active partnership between patients, carers and members of the public with researchers’ [51]. Whereas some models use the terms ‘participation’ and ‘involvement’ synonymously [15, 26], participants in the present study rejected ‘participation’ as a type of involvement, consistent with interpretations elsewhere in the literature [56]. However, the YROG commented that there can be a blurring of boundaries between participation and involvement, and that activities such as focus groups can be experienced as being ‘done to’ by one participant and ‘done with’ by another.

There were both similarities and differences in the features of the MYPI produced in the present study with existing models. The MYPI developed here has parallels with Darney et al. (2019)'s model [57] which proposes four levels of youth participation—‘participation’, ‘consultation’, ‘partnership’ and ‘youth‐led’ but in the present study ‘partnership’ is separated into ‘coproduction’ and ‘coresearch’. The ‘Ladder of coproduction’ [25] includes both consultation and coproduction, in the same order as in this MYPI, but peer‐led involvement, a higher order of involvement, is absent from the ‘Ladder of Coproduction’, as it is with McCain Model of Youth Engagement [29]. In Hart's ‘Ladder of Participation’ [26], ‘Child initiated and directed’ involvement (Level 7) represents a lower ‘rung’ than ‘Child initiated, shared decisions with adults’ (Level 8). Conversely, in this MYPI, ‘young person or peer‐led’ research (the equivalent of Hart's Level 7) was placed higher than coproduction (the equivalent of Hart's Level 8), as research ‘done by’ young people represents a higher level of involvement than research ‘done with’ young people. ‘Communities of involvement’, where young people are proactive in identifying opportunities, to get involved and to support other young people to engage in research, was the highest level of involvement in the MYPI here but does not feature in other models. A similar concept appears, and is described, in Swist et al.'s [58] Communities of Practice as ‘distinct from involving young people in individual research projects and is aimed at understanding how to embed engagement with young people in ongoing processes of health research and translation’ [58]. The involvement levels to the right of the continuum in this MYPI align with the principles of ‘Participatory Action Research’ where young people play an active role in the conduct of research for example as coresearchers [22]. Thus, the MYPI presented here, complements other models, drawing together elements common in existing approaches but providing a broader, and more comprehensive, picture of involvement. It is more detailed and practical than many models which are conceptual in nature. It brings up to date the picture of the involvement landscape in the context of young people's mental health research which has evolved significantly since some previous models were created.

### 4.2 Diversity and Representation

Panellists agreed that the involvement of *diverse* young people in mental health research was poor. This is significant as research into the quality of young people's involvement, particularly for diverse young people, is severely limited. The YROG felt that young people were often unaware that there are opportunities to be involved in mental health research and that, for those who were aware, complex language, lengthy documents and the time required to develop the necessary knowledge and skills to be involved as an equal partner could be a barrier, especially for under‐represented groups. The NIHR INCLUDE project [50, 59] provides guidance on improving inclusion in participation in clinical research. Although the context for the present study is different, there are nevertheless relevant comparisons. The NIHR INCLUDE project, similar to the present study, used consensus building with stakeholders, to develop guidance. They advise using the term ‘under‐served’ for groups which are less included in research, suggesting that, ‘the term “under‐served” reminds us … [that] the lack of inclusion is not due to any fault of the members of these groups … in a way that alternative terms such as “under‐represented” do not’ [50]. Conversely, in the present study, the term ‘under‐served’ was only preferred by one participant, compared to 14 participants selecting ‘under‐represented’. Terminology is consequential and sensitive, as certain terms can be stigmatizing (e.g., ‘hard to reach’) [60] or imply that lack of involvement is the responsibility of those not involved [33, 61], so identifying a term which the majority of participants (including the majority of young experts) agree with is significant, despite consensus not being reached. Participants in the present study agreed a definition of under‐representation in the context of involvement of young people in mental health research which emphasised diversity and representation of young people from different backgrounds and demographics, such as marginalised groups. In contrast to the reference to ‘population estimates’ [50] specified in the NIHR's description of under‐served, the definition agreed in the present study aligns more closely with the concept of ‘scientific equity’ where ‘all relevant populations, including those who have been historically disadvantaged, are properly included’ [62]. The YROG agreed with the term and definition of panel experts and also emphasised the need to avoid language that could imply blame on those who are under‐represented. They considered under‐representation to be a complex concept, noting that individuals have multiple aspects to their identity, any of which may not fit with the majority. This is reflected in a recently proposed Diversity Minimal Item Set which takes an intersectional approach to diversity [63] and recognises that different aspects of a person's identity interact and can exacerbate disadvantage.

Drawing on their experience, panellists in the present study reached consensus on groups under‐represented in young people's involvement in mental health research. Given the lack of data reported on the characteristics of young people involved in mental health research in the literature these agreed groups provide a focus for researchers recruiting young people to involve in their research. Participants in the present study reached consensus on 17 categories of data that should be collected from young people involved. These extend both the characteristics suggested in American Psychological Association (APA) guidance; ‘age; sex; ethnic and/or racial group; level of education; socioeconomic, generational or immigrant status; disability status; sexual orientation; gender identity; and language preference’ [64] and the nine characteristics recommended in the Diversity Minimal Item Set [63]. Collecting and reporting on a broad range of diversity domains helps understand the nature of the sample and drives ‘diversified innovations’; ‘evidence‐based discoveries, emerging from a systematic description of differences’ [63] as well as being useful for future meta‐analytic studies [63, 64]. However, caution is advised that only data pertinent to the research topic should be kept and reported [65]. In reality at present, often not even basic levels of demographic data are collected or reported [13].

In the present study, participants agreed that young people were under‐represented in all roles and in some stages of the research process, although some believed that involvement was potentially not necessary in data collection and analysis. There are differing views in the literature, with some believing that a flexible approach should be taken based on the needs of the particular study and the preferences and strengths of the young people involved [66], whereas others believe that representation is important at all stages and levels [67]. In practice, the literature suggests that young people's involvement in different stages of the research process is variable with fewer studies showing young people involved in analysis, evaluation and dissemination of research [24].

Overall, the areas of consensus in the present study offer new and valuable expert perspectives for researchers considering when and how to involve a diversity of young people in their research. For example, through reframing definitions of under‐representation, considering demographic data to collect and through thinking about the different roles that young people can play. This is important as existing recommendations for improving involvement often focus either on children and young people or diverse groups but not both.

Finally, the failure to reach consensus in some areas illustrates the lack of unanimity, even amongst experts, on issues such as the distinction between participation and involvement, where young people should be more involved in research and the most appropriate terminology to describe those who are under‐represented.

### 4.3 Limitations

This Delphi study included three rounds a priori, rather than continuing until consensus was reached, to encourage participation by ensuring that participants were aware of the time commitment involved before consenting to take part. Had the study continued until consensus was reached in all aspects, a more definitive view of experts could potentially have been reported. However, failure to reach consensus is, in itself, considered a valid and insightful finding [48]. A further potential limitation was that some experts were specialists in research with particular groups of young people and therefore their knowledge of diversity in involvement more broadly may have been limited and potentially affected participants' responses on which groups of young people are under‐represented. The consensus‐building approach reduces this bias by requiring a majority of experts to agree. By considering the three types of experts as a single panel It is also possible that a different set of results would have been obtained if experts had been considered in their three separate groups. However, this was mitigated by analysing data by each expert group to ensure consistency across different expert groups and through the nature of the Delphi process itself which allowed participants to validate findings and develop consensus through the rounds. The consultation with young people (through the YROG) in this study was highly valuable but the scope of their input was limited by budget constraints. Finally, the study was conducted in a UK context with all participants being based in the United Kingdom, limiting its generalisability to different geographical contexts where young people's involvement may be more, or less, well established.

### 4.4 Conclusion

This study contributes to the fields of young people's mental health research and of PPI, by offering a detailed, practical and context‐specific MYPI of the different types, activities and roles that young people can have in mental health research, both existing and emerging. It provides expert ratings of involvement of young people, and specifically of diverse young people, in the UK context. The findings demonstrate that further work is needed to improve diversity in the involvement of young people in mental health research, which should start with more detailed reporting and a better understanding of why some groups are under‐represented. This is particularly pressing given the increase in mental health problems faced by young people, coupled with the significant proportion of young people who are not accessing professional mental health support. This paper highlights the importance of diversity in involvement, pinpoints the gaps and provides a useful reference guide for researchers planning to undertake involvement activities with young people. This work also reinforces that having a shared understanding and clarity over terminology and definitions are fundamental to improving reporting, building the knowledge base and contributing to improvements in the area of involvement. Future research should further explore the diversity of young people involved in mental health research based on the demographic categories identified in this study, and investigate barriers to involvement for under‐represented young people.

## Author Contributions

**Rachel Perowne:** conceptualization, investigation, funding acquisition, writing–original draft; methodology, formal analysis. **Sarah Rowe:** writing–review and editing, supervision, methodology. **Leslie Morrison Gutman:** writing–review and editing, supervision, methodology.

## Conflicts of Interest

The authors declare no conflicts of interest.

## Supporting information

Supporting information.

Supporting information.

Supporting information.

## Data Availability

The data sets generated during and/or analysed during the current study are not publicly available for ethical and sensitivity reasons. Deidentified summaries of some data may be available on reasonable request from the corresponding author.
