# Supplementary material for: Understanding and Defining Young People's Involvement and Under‐Representation in Mental Health Research: A Delphi Study
Source: Health Expect. 2024 Jun 14;27(3):e14102. doi: 10.1111/hex.14102 (PMC11176595; doi:10.1111/hex.14102)
Supplement: Supplementary file 1 — Supporting information. [file HEX-27-e14102-s002.docx]

**Appendix 1: Round 1 Questionnaire**

**Diversity and representation in young people's involvement in mental health research**

**Thank you**

Thank you for agreeing to participate in this three-round Delphi survey. You are one of around 18 participants. Each participant has been selected on the basis of their experience or expertise of involvement of young people in mental health research; either as an academic researcher, a practitioner working in Patient and Public Involvement or Co-Production or as a young person who is an expert through their experience of being involved in mental health research (for example as a member of a Young Persons Advisory Group or through Patient and Public Involvement activities).

**Purpose of the study**

Young people's mental health is a growing concern in the UK. In 2022, 22% of young people aged 16-24 were thought to have a 'probable mental health disorder'.

However, young people experience mental health differently. For example, evidence suggests that:

- young people with long term physical health conditions are more than twice as likely to have a probable mental health disorder than those without
- lower household income is associated with increased adolescent mental health problems
- young people from minoritised ethnic groups are more likely to enter the mental health system through compulsory rather than voluntary routes
- sexual minority (eg lesbian, gay or bi-sexual) young people are more likely to experience depressive symptoms than sexual majority young people.

Involving young people of different identities and backgrounds in mental health research is critical to designing inclusive services and support that meet the needs of all young people. Involvement can take many forms, from 'passive', where young people are participants in a study, through to more 'active' where young people provide an advisory role or are co-producers or co-researchers.

The purpose of this Delphi study is to seek consensus, from a range of experts, about diversity within young people's mental health research, at different levels of involvement.

**Process**

There will be three rounds of data collection involved in the study. Each round will involve you completing a questionnaire which should take on average no more than 20 minutes to complete. If there are any questions you can't answer, that's fine, please just leave them blank. There are no right or wrong answers - all your views are valued. After each round you will receive an anonymised summary of the results. The aim, by the end of the third questionnaire will be to have built a consensus on which groups of young people, if any, are under-represented in the involvement of young people in mental health research. The first round comprises of 26 questions - some tick box and others open ended. You are asked to complete and submit this questionnaire by 21 June 2023.

**About you**

Please note that your personal details will not be shared outside the research team, including to other participants. This in‐ formation is being gathered so we can understand the identity and diversity of our expert participants. No other participants will be able to identify your individual responses and your details will not be referred to in any presentations of the results.

1. Name
2. Organisation or affiliation (eg g University, NHS Trust or Young People's Advisory Group)
3. Ethnicity
4. Gender
5. Type of expert (tick all which apply)
   - Young person with experience of being involved in mental health research
   - Co-production or involvement professional with experience of working in young people’s mental health research
   - Academic researcher with experience of young people’s mental health research
6. Please outline your experience and/or expertise in involvement of young people in mental health research. You may wish to include (where applicable):
   - job title
   - papers you have published
   - projects or studies you have been involved in and the role you played and
   - years' of experience

**What do we mean by involvement?**

One of the difficulties in investigating young people's involvement in mental health research is that different terms are used for different types of involvement.

In no particular order, from your direct experience, or based on your expertise, please list the different types of involvement you are aware of that young people could have in mental health research. For each type please provide:

1. A definition or description of this type of involvement

2. The sorts of activities involved

3. The benefits of this type of involvement to young people's mental health research

Examples might include, but not be limited to, Young People's Advisory Groups, Co-production and Co-research.

Note: Please use a separate box for each type of involvement and do not feel you have to use all boxes

1. Type of involvement 1 (including definition/description, types of activities and benefits
2. Type of involvement 2 (including definition/description, types of activities and benefits)
3. Type of involvement 3 (including definition/description, types of activities and benefits)
4. Type of involvement 4 (including definition/description, types of activities and benefits)
5. Type of involvement 5 (including definition/description, types of activities and benefits)
6. If you would like to include additional types of involvement please feel free to add them here

**The current state of involvement**

1. Based on the types of involvement you have identified above and from your experience, overall how well do you think young people are currently involved in mental health research in the UK? From 1 – 5 where 1 is Very poor and 5 is Excellent
2. Please explain your rating below - including whether you think representation is better in some types of involvement than others

**Defining diversity and under-representation**

1. when we talk about young people being under-represented in mental health research what do you understand by this? How would you define this?
2. Is under-representation the most appropriate term to describe those whose voices may not be adequately heard in mental health research? If not, what other words or phrases might be more appropriate?

**Diversity and representation in young people's mental health research**

1. Thinking more specifically about diversity and under-representation, overall, how well do you think young people from diverse backgrounds and identities are currently involved in mental health research? From 1 – 5 where 1 is Very poor and 5 is Excellent
2. Please explain your answer above
3. Following on from questions 15 and 16, what groups or identities of young people in particular, if any, do you feel are under-represented in research into young people's mental health?
4. Thinking about the different stages of the research process, please describe at which stages of the research process, if any, you feel there is under-representation or a lack of diversity in young people's involvement. You may want to identify some of the following stages in the research process but please do not feel limited by this and add free text answers in the box below:
   - Setting research priorities (ie deciding what research should be conducted)
   - Commissioning and securing funding (eg putting bids together for funding or awarding funding to projects)
   - Designing and planning research studies
   - Data collection (eg conducting interview, trials, questionnaires etc)
   - Data analysis (eg coding data, finding themes, reviewing data)
   - Dissemination (ie communicating the results of the research)
   - Knowledge translation (ie putting the findings of research in to practice)
   - Evaluation (ie reviewing research and assessing how well it worked)
   - Other
5. Please explain more about your answer above
6. At what levels of involvement do you think there is under-representation or lack of diversity (if any)? For example, young people as advisors, co-researchers, co-producers and any other types of involvement you have identified
7. What socio-demographic information should we collect from young people involved in mental health research? The list below includes some basic options for you to select but we want to think about representation and identity in the widest sense so please add any additional data you think is important in the free text box below
   - Age
   - Sex
   - Gender
   - Ethnicity
   - Religion and belief
   - Disability
   - Neurodiversity
   - Socio-economic status
   - Carer status
   - Other - please expand below
8. Please add any categories and explain your answer above
9. What ways have you used or seen to recruit a diverse range of young people of different identities to be involved in mental health research?
10. Do you consider yourself to be from a group that is under-represented in mental health research? If so which aspect of your identity does this apply to?

**Thank you!**

Thank you very much for taking the time to complete this questionnaire. You will receive your second round questionnaire for completion in the next 4 weeks or so. The next questionnaires will include more closed questions and so be quicker to complete.

If you have any questions or would like to discuss this questionnaire or the study in more detail please contact Rachel Perowne at rachel.perowne.19@ucl.ac.uk
