# Supplementary material for: Understanding and Defining Young People's Involvement and Under‐Representation in Mental Health Research: A Delphi Study
Source: Health Expect. 2024 Jun 14;27(3):e14102. doi: 10.1111/hex.14102 (PMC11176595; doi:10.1111/hex.14102)
Supplement: Supplementary file 3 — Supporting information. [file HEX-27-e14102-s003.docx]

**Appendix 3: Types of involvement, definitions identified by expert panellists with selected quotes to illustrate**

| **Type of Involvement** | **Involvement?** | **Definition provided in round 3** | **Selected quotes (from all rounds)** |
| --- | --- | --- | --- |
| **Type 1: Young People's Advisory Group** | Yes | A group or panel for consulting young people, sometimes with lived experience of mental health issues, convened to inform research activity. Young people share their views and experiences as well as providing advice and feedback to researchers to make meaningful contributions to improve research and inform decision making, either on a project specific, or regular and ongoing, basis.   Activities include young people:   - Attending, or chairing, meetings (such as workshops and focus groups) or giving views remotely (eg by email) - Contributing to the development of the research process from defining research questions and priorities, suggesting improvements to the research process through to supporting the dissemination of research findings - Reviews of, or co-design of, study materials and documents, such as research proposals, recruitment strategies, participant information sheets, interview questions and other communication materials | *'This involves young people making meaningful decisions about the strategic direction, and the specific attributes and details of the research project’.* (P25R1) |
| **Type 2: Co-production (including co-design and co-creation)** | Yes | A process of active involvement and collaboration with young people, either as a collective or individually, throughout the research process (from inception and creation to implementation and dissemination) – starting from scratch/a blank page. Following the 5 co-production principles, young people are equal and reciprocal partners, gaining benefit themselves and working alongside researchers, having a say about how the research is done and being part of decision making.   Activities include young people:   - Attending steering groups, meetings, workshops or focus groups to discuss the research and where young people can share their ideas, thoughts and feedback to inform the research design - Sharing details of the study with communities and recruiting participants - Working with researchers on the design or production of ideas and materials for a study - Approving studies and study materials - Having a say in data analysis, conclusions and recommendations | *‘As I understand it: Co-design is an attempt to define a problem and then define a solution; co-production is the attempt to implement the proposed solution; co-creation is the process by which people do both’ (P7 R3)*  *‘Co-creation is still different to co-design IMHO [in my humble opinion], as co-design is starting from that blank piece of paper’ (P2 R3)* |
| **Type 3: Consultation or advisory role** | Yes | Young people’s views and expertise are sought in relation to a project either on a one-off or ongoing basis. Input is often limited in scope and the young people’s advice may or may not be acted upon. In this sense the young people are not equal and reciprocal partners in the research in the same way as in co-production.   Activities include:   - Young people attending and providing input a dedicated workshop, focus groups , 1:1 interviews or feedback elicited through an existing group (such as a Young People’s Advisory Group) - Young people reviewing and commenting on study materials such as Participant Information Sheets or on outputs from data collection or analysis - Feedback provided to the young people on their contributions | *‘I think you can be an advisor and an equal/reciprocal partner, they're not mutually exclusive… It's more about the ethos in which you conduct your work, if the advisory board has been convened as a box ticking exercise or in a tokenistic way, then you may be motivated to ignore their contributions.’* (P27R3)  *‘I am just not sure about feedback falling into this category. I think that being given feedback helps young people expand their knowledge of mental health and also motivates them to stay involved. It is just that it is not a direct contribution from young people but feedback from researchers to young people as advisors.’* (P22R3) |
| **Type 4: Co-research/peer-research** | Yes | Young people are very actively involved in key stages of the research process, bringing lived experience to the research and conducting research themselves, with the supervision of a senior researcher. A slightly more involved role than co-production. This could be done through an internship, bursary or a voluntary or paid opportunity.   Activities include young people working with researchers in:   - Designing studies - Contributing to literature reviews - Collecting data (eg interviewing) - Data analysis - Writing up findings from research | *‘Maybe just highlight more that this is about conducting research, rather than advising on/developing.’* (P7R3) |
| **Type 5: Patient and Public Involvement** | Yes | An umbrella term for co-production and co-design which involves activities that add value or knowledge to the project through seeking the advice and opinion of young people, who would be affected by the research, on a piece of mental health research.   Activities include young people:   - Creating advertising materials - Attendance at focus groups - Providing a written response to a call for feedback - Reviewing materials to ensure they are accessible for the audience - Supporting the dissemination of research work - Being part of a steering group | *‘dont think PPI necessarily covers co-production only. think it also covers more consultative processes.’* (P24R3)  *‘I would say it is broader than this, beyond co-production and co-design. Co-production and co-design definitely fall into it and are great examples of PPI (something to aim for even), but PPI can also be more light touch.’* (P1R3)  *‘I don't really see 'providing a response for feedback' as part of involvement and more participation.’* (P4R3)  *‘I would argue it is understood by those in the field that PPIE is the umbrella term for any and all forms of involvement and engagement (from consultation, coproduction etc)’* (P7R3)  *‘If only attending focus groups & creating materials then IMHO those people would be PPIE (engagement)*  *If doing All of the above, then yes PPI’.* (P2R3) |
| **Type 6: Young Person/peer led involvement and research** | Yes | Young people, with lived experience, take charge of all aspects of the research process, leading, having ownership, meaningful involvement and making decisions across all stages of the research process. Researchers provide supervision and support but there is reciprocity and power is equally shared with the young people.   Activities include young people:   - Setting research questions - Designing study materials and forms - Being involved in leading the research and involvement process - Involved in collecting and analysing data, identifying and disseminating findings | *‘They would probably need to secure funding as well if necessary.’* (P12R3) |
| **Type 7: Reviewer** | Yes | Young people review or evaluate research documents, potentially as part of a panel of reviewers, and provide feedback and recommendations.  Activities include:   - Reviewing and commenting on research documents - Reviewing or evaluating grant/funding applications | *‘Yes but also add like reviewing in the sense of judging for awards.’* (P4R3) |
| **Type 8: Young people’s involvement network/community of practice** | Yes | A youth focused approach to involvement which can make different types of involvement more meaningful. Networks or communities of young people who have an interest in being involved in research are developed. Some networks act as a vehicle for young people to find out about opportunities or they can be groups that meet regularly for young people to direct the involvement process and decide which research questions should be addressed, and types of involvement to engage in. They are a resource for young people to learn about new opportunities to get involved.   Activities can include:   - Regular meetings for young people to discuss involvement and/or involvement in research opportunities - Young people responding to calls for involvement | *‘Also, offer training and development for young people involved, beyond just involvement in research.’* (P7R3) |
| **Type 9: Young People as participants** | No | Young people participate in research, as a study subject or service user.   Activities include:   - Completing questionnaires - Being part of experiments and completing tasks - Undertaking tests such as neuroimaging - Taking part in a research focus group or interview | *'This is not involvement. Patient and public involvement sees research being carried out ‘with’ or ‘by’*  *members of the public, rather than ‘to’, ‘about’ or ‘for’ them’.* (P23R2) |
| **Type 10: Young Persons' Representative on a scientific committee** | Yes | For example, young people being a representative on a thesis committee or steering committee.   Activities include:   - Providing comment on findings and conclusions - Asking for young people’s views on the framing of research questions. | *‘Maybe YP can also comment on the importance of findings or research questions, according to their experience.’* (P12R3) |
| **Type 11: Involvement ambassadors** | Yes | Young people (from communities who are all too often easily ignored) are set up as involvement champions in health care research.  Activities include:   - being trained in health care research and involvement - leading/co-ordinating involvement activities within their own communities. - running workshops and meetings - acting as support/first contact for other young people who may usually be wary or just unlikely to usually get involved in both PPI and health care research more generally. | *‘I would say this falls under the Young people’s involvement network/community of practice - with a youth-led approach to involvement. It is a common role under this PPI approach, to ensure that the types of involvement the group engages in are meaningful and every voice is represented’* (P17R2) |
| **Type 12: Dissemination** | No | Feeding back results of research to participants and the broader community  Activities include:   - young people attendings webinars, talks, workshops etc | *‘Plus receiving resources or summaries of the research they were involved in.’* (P27R3) |
